# Soybean Oil Bodies as a Milk Fat Substitute Improves Quality, Antioxidant and Digestive Properties of Yogurt

**DOI:** 10.3390/foods11142088

**Published:** 2022-07-14

**Authors:** Nianxu Dou, Rongbo Sun, Chengcheng Su, Yue Ma, Xuewei Zhang, Mengguo Wu, Juncai Hou

**Affiliations:** College of Food Science, Northeast Agricultural University, Harbin 150030, China; nxdou980223@163.com (N.D.); surbo0215@163.com (R.S.); sucheng971128@163.com (C.S.); myue09418@neau.edu.cn (Y.M.); 17703647962@163.com (X.Z.); wmg280586574@163.com (M.W.)

**Keywords:** soybean oil bodies, yogurt, quality characteristics, vitro digestion, antioxidant

## Abstract

In this experiment, the effect of replacing milk fat with soybean fat body (25%, 50%, 75%, 100%) on the quality, antioxidant capacity and in vitro digestive characteristics of yogurt was investigated while maintaining the total fat content of the yogurt unchanged. The results showed that increasing the substitution amount of soy fat body for milk fat had little effect on the pH and acidity of yogurt during the storage period, while the physicochemical properties, degree of protein gel network crosslinking, saturated fatty acid content, PV value and TBARS value of the yogurt significantly decreased (*p* < 0.05). Meanwhile, protein content, solids content, unsaturated fatty acid content, tocopherol content and water holding capacity significantly increased (*p* < 0.05). Flavor analysis revealed that yogurts with soybean oil bodies were significantly different when compared to those without soybean oil bodies (*p* < 0.05), and yogurt with 25% substitution had the highest sensory score. After in vitro digestion, the free fatty acid release, antioxidant capacity and protein digestibility of soybean oil body yogurt were significantly higher (*p* < 0.05). The SDS-PAGE results showed that the protein hydrolysis of the soybean oil body yogurt was faster. Therefore, the use of an appropriate amount of soybean oil bodies to replace milk fat is able to enhance the taste of yogurt and improve the quality of the yogurt.

## 1. Introduction

Yogurt is a gelatinous product that takes raw cow (sheep) milk or milk powder as raw material that, after heat treatment or concentration, is fermented using lactic acid under the action of *Streptococcus thermophilus* and *Lactobacillus delbrueckii subsp. bulgaricus* [1]. Yogurt can be classified according to different methods and organizational states. The most common yogurts on the market are stirred yogurts and gelatinous yogurts. These two types of yogurt come in a variety of forms, and most of them cater to buyers by adding ingredients such as grains or fruits to meet their taste and nutrition needs [2]. Compared to milk, yogurt lasts longer and contains a variety of good gut bacteria [3]. Yogurt can be consumed by people with lactose intolerance without suffering from diarrhea or other symptoms, because part of the lactose in the milk is converted into lactic acid under the action of microorganisms during the fermentation of the yogurt [4]. At the same time, the probiotics found in yogurt can help maintain the ecological balance of intestinal flora, regulate the immune response, and slightly reduce the pH value of gastric juice, so consumers are able to decreased concerns regarding low gastric juice secretion and pathogen transmission. Yogurt also contains active lactase, which aids digestion of lactose [5,6]. Data show that the sales volume of yogurt products in China increased from 45.6 billion in 2012 to 220 billion in 2022, representing a growth rate of 9.2%. The proportion of the total dairy market accounted for by yogurt products increased from 20% in 2014 to 36% in 2019. It is expected to rise further to 42.2% by 2024. With the increasing demand for yogurt, it is very important to produce high-quality, differentiated, and functional yogurt. In the future yogurt market, yogurt with targeted characteristics will become a mainstream product, and plant-based yogurt may become a new star in the future [7].

Soybean oil bodies (SOBs) are composed of Triacylglycerols (TAGs) and coated by a single phospholipid membrane. At the same time, Oleosin, Caleosin and Steroleosin are embedded on the surface of the phospholipid membrane to form complete, independent, triacylglycerol-rich discrete membrane organelles with diameters ranging from 0.4 μm to 2.0 μm, which can provide energy for seed growth, development, and metabolism [8,9]. SOBs are mainly composed of neutral lipids (92~98%) (*w*/*w*), and also contain small amounts of protein and phospholipids [10]. Phospholipid molecules of water head are exposed in the cytoplasm, and therefore SOBs have a certain degree of hydrophilicity. The protein covering on the surface of SOBs prevents the phospholipase from interacting with the phospholipid layer of the outside, enhancing the stability of the SOBs, inhibiting SOB agglomeration or fusion, and preventing the oxidation of SOBs [11]. At present, the research on SOBs in the field of food is mainly focused on their stability and potential applications as food components. For example, Wu et al. [12] studied the effects of different ι-carrageenan concentrations on the bioavailability of fatty acids and Vitamin E in SOBs, and found that with increasing ι-carrageenan concentration, the bioavailability of total fatty acids and Vitamin E in SOBs gradually decreased. Karkani et al. [13] mixed a natural oil body emulsion with green tea extract as a base for functional drinks and found that natural oil bodies combined with green tea polyphenols to form an unstable product, but this could be improved by the addition of a small amount of carrageenan. Mantzouridou et al. [14] used corn germ oil bodies to replace milk fat in yogurt formula and found that oil bodies interacted with the milk protein network structure and offered better gel strength than skim milk. However, the practical application of SOBs in products is still lacking. Therefore, it is of great significance to use SOBs as supplementary ingredients in products. Studies have found that, compared with animal oils, SOBs contain higher contents of unsaturated fatty acids and tocopherol, and are rich in phospholipids and phytosterols [15]. Therefore, it is very important to explore the effects of using different percentages of SOB as a milk fat substitute on yogurt quality, antioxidant capacity, and digestion characteristics.

In this experiment, the physicochemical properties, rheological properties, fatty acid content, tocopherol content, flavor, microstructure, PV value, and TBARS value of yogurt in which milk fat was replaced with different quantities of SOBs (25%, 50%, 75%, 100%) were determined under conditions in which the total fat content of the yogurt remained unchanged. The antioxidants, free fatty acid release, and protein digestibility of yogurt with different degrees of substitution in simulated human oral and gastrointestinal digestive systems were studied. The results showed that it is possible to improve the nutritional value of yogurt, laying the foundation for the development and utilization of SOBs.

## 2. Materials and Methods

### 2.1. Materials and Chemicals

Soybeans were provided by Northeast Agricultural University. Whole milk (3.2% protein and 3.8% fat) and skim milk (3.2% protein) were purchased from Yili Industrial Group Co., LTD. (Hohhot, China), sugar was bought from Carrefour Supermarket in Harbin, and starter cultures (*Lactobacillus delbrueckii subsp. bulgaricus*, *Streptococcus thermophilus*) were purchased from Danisco Group (Guangzhou, China). Copper sulfate, potassium sulfate, sulfuric acid, boric acid, sodium hydroxide, anhydrous ethanol, ethyl ether, petroleum ether, cobalt sulfate seven water, methanol, sucrose, boron trifluoride, n-heptane, sodium chloride, sodium sulfate, n-hexane, isopropyl alcohol, isooctane, n-butyl alcohol, potassium thiocyanate, isopropyl benzene, trichloroacetic acid, chloroform, methyl red indicator, indicator bromocresol green, methylene blue indicator and phenolphthalein, all designated as analytically pure, were purchased from Comere Chemical Reagent Company (Tianjin, China). Thiobarbituric acid, SDS, PBS, Tris, Coomassie Bright Blue G-250, DPPH and ABTS, all analytically pure, were purchased from Sigma Company (Shanghai, China). Nile red and Nile blue were analytically pure, and were purchased from Amresco (Shanghai, China). Trypsin, pepsin, and lipase were purchased from Yuanye Biotechnology Co., LTD. (Shanghai, China). Bovine serum albumin was purchased from Jingchun Industrial Co., LTD. (Shanghai, China). MRS AGAR medium and MC AGAR medium were purchased from Best Biotechnology Co., LTD. (Hangzhou, China).

### 2.2. Preparation of SOBs

The extraction of SOBs was performed based on the method of Zhou et al., with some improvements [10]. The soybean was cleaned several times, soaked in deionized water at 1:5 *w*/*v* for 15 h, and then mixed with 20% sucrose solution (1:5 *w*/*v*) and ground in a tissue masher for 3 min. Filtrate was obtained by filtering with 4 layers of degreased gauze. The filtrate was centrifuged (10,000× *g*, 4 °C, 30 min) to collect the upper cream. The suspended solids were then placed in 20% sucrose solution (1:3 *w*/*v*) and centrifuged (10,000× *g*, 4 °C, 30 min). The process was repeated three times, and the last washing medium used was deionized water, and SOBs (26.05% fat, 9.12% protein, 60.76% moisture) were obtained. The SOBs were heat treated in a 95 °C water bath for 20 min.

### 2.3. Preparation of SOB Yogurt

The total fat content of yogurt without SOBs was 3.57 g/100 g. The SOBs were used to replace milk fat (hereinafter referred to as “substitution amount”) in proportions of 0% (control group), 25%, 50%, 75% and 100% of the total fat, and whole milk or skim milk was mixed with SOBs to make SOB yogurt with a constant total fat content, before the addition of white granulated sugar amounting to 6% of the total weight. The mixture was homogenized at 20 Mpa, pasteurized (65 °C, 30 min), cooled to 42 °C after heat treatment, inoculated in a super clean workbench with an inoculation amount of 0.01% of the total mass, and then fermented in a constant-temperature incubator (42 °C, 5 h). After fermentation, the samples were transferred to the refrigerator at 4 °C and cooked for 24 h.

### 2.4. Physicochemical Properties of SOB Yogurt

The AOAC [16] was used to measure the protein, fat, total solids, acidity, moisture content, and pH values of the SOB yogurt. The contents of fatty acids were determined by gas chromatography-mass spectrometry (Nexis GC-2030, Shimadzu, Japan) [17]. L*, a*, b*, ΔE were read directly using a chroma analyzer (ZE6000, Nippon Denshoku, Tokyo, Japan). L* values represent brightness, and a* and b* values represent chroma. The flavor of the SOB yogurt was measured using an electronic nose. Principal component analysis was performed on 10 groups of representative data after repeated measurements of each sample.

### 2.5. Water Holding Capacity of SOB Yogurt

Appropriate modifications were made according to Ercilicura et al. [18]. Weigh 20 g yogurt samples in a centrifuge tube (32 mm × 115 mm), pour off the supernatant after centrifugation (8000× *g*, 20 °C, 10 min), and place the centrifuge tube upside down for 10 min to weigh it, and calculate the water holding capacity according to the following formula: where Q denotes the water holding capacity (%), W_1_ denotes the mass of the sample before centrifugation (g), and W_2_ denotes the mass of the sample after centrifugation (g).

$$Q=\frac{W_{1}-W_{2}}{W_{1}}\times 100$$

### 2.6. Tocopherol Content of SOB Yogurt

Appropriate modifications were made according to Bertolin et al. [19]. First, 0.5000 g of the freeze-dried yogurt sample was accurately weighed and placed into a brown volumetric flask. N-hexane was added to dissolve, and ultrasonic extraction was conducted in an ice water bath for 10 min. After centrifugation (8000× *g*, 4 °C, 10 min), the extract was blow-dried with nitrogen, and 1 mL isopropyl alcohol was added to dissolve the oil component. Then, filtration was performed using an organic filter membrane system (pore size 0.22 μm). The filtrate was analyzed using high-performance liquid chromatography, the peak area was measured, and the concentration was calculated on the basis of the standard curve using the external standard method. 

HPLC conditions: SB-C18 column (9.4 × 250 mm, 5 μm); fluorescence detector, excitation wavelength 290 nm, emission wavelength 340 nm; column temperature 35 °C. The mobile phase was methanol-water (volume percentage: 96:4). The flow rate was 0.8 mL/min. The injection volume was 10 μL.

### 2.7. Texture Characteristics of SOB Yogurt

The texture of the SOB yogurt was determined using a texture analyzer (TA-XT Plus, SMATA, London, UK). The specific test parameters were as follows: A/BE-D35 mold probe, pre-test speed 1.0 mm/s, test speed 1.0 mm/s, post-test speed 1.0 mm/s; the induction force was set as 5 g, and the probe running was 10 mm.

### 2.8. Rheological Properties of SOB Yogurt

The rheological properties were determined according to the method proposed by Jiang et al. [20]. The sample was tested at (25 ± 0.5) °C on the plate (diameter 60 mm) of a rotary rheometer (MARS40, Thermo, Massachusetts, America), and the gap between the plate and the bottom surface was set at 1 mm.

First, the frequency was fixed at 1 Hz, the strain was scanned from 0 to 50%, and the linear viscoelastic range strain of the sample was determined to be 0.6%. Sample test: frequency scanning, strain fixed at 0.6%, frequency scanning from 0.1–10 Hz. Shear scanning was performed from 0 to 100 s^−1^, with a scanning time of 2 min. The above tests were all performed within the online elastic range.

### 2.9. PV and TBARS Values of SOB Yogurt 

Appropriate modifications were made according to the methods of Su et al. [21]. First, 1.5 mL isooctane/isopropyl alcohol (2/1, *v*/*v*) was added to a 0.80 g yogurt sample, followed by vortexing (3 times, 10 s duration) and centrifugation (2000× *g*, 2 min). Then, 0.5 mL organic phase was added to 3 mL methanol/n-butanol (2/1, *v*/*v*). Add 20 μL thiocyanate (3.94 mol/L) and 20 μL 0.072 mol/L Fe^2+^, and react with light for 20 min. Then, measure the absorbance at 510 nm. Use isopropylbenzene as the standard curve to determine the PV value.

A 0.80 g yogurt sample was mixed with 1 mL trichloroacetic acid solution (10%, *w*/*v*) and 2.5 mL thiobarbituric acid solution (1%, *w*/*v*), boiled for 30 min, and cooled to room temperature for 10 min. The cooled mixture was mixed with 0.5 mL chloroform and centrifuged (4000× *g*, 15 min). The supernatant was a red thiobarbituric acid reaction product, and the absorbance was measured at 532 nm. The content of TBARS was measured according to the molar extinction coefficient 152,000 M^−1^cm^−1^.

### 2.10. Microstructure of SOB Yogurt

The microstructure was determined according to the methods of Zhou et al. with some modification [22]. Laser scanning confocal microscopy (Deltavision OMX SR, GE, Fairfield, CT, USA) was used for observation. First, 1 mL sample was diluted 3 times and added to 80 μL of 0.02% Nile Red and 0.1% Nile Blue A staining solution for preparation. The excitation wavelength was 488 nm and 633 nm.

### 2.11. Sensory Evaluation of SOB Yogurt

Sensory evaluation was performed according to the methods of Marand et al. with appropriate modifications [23]. Fifteen laboratory students were randomly selected to participate in the sensory evaluation. The samples in each group were aged for 24 h and placed in disposable plastic cups with three kinds of English alphabet coding, and then left to stand until the sample temperature had risen to 8–10 °C for sensory evaluation. Sensory evaluation involves five indicators: appearance, taste, bean smell, acidity, and overall evaluation. The scoring standard is 9 points, whereby the higher the scores for appearance, taste and overall evaluation, the better the rating on these dimensions. The higher the bean smell and acidity, the higher the degree.

### 2.12. In Vitro Simulated Digestion of SOB Yogurt

Simulated saliva (SSF) was prepared as follows: sodium lactate 0.146 g/L, ammonium nitrate 0.328 g/L, potassium chloride 0.202 g/L, sodium chloride 1.594 g/L, adjusted to pH 6.8.

Simulated gastric juice (SGF) was prepared as follows: glucose 0.65 g/L, sodium dihydrogen phosphate 0.27 g/L, ascorbic acid 0.0176 g/L, potassium chloride 0.82 g/L, sodium chloride 2 g/L, pepsin 3.2 g/L, adjusted to pH 2.0.

Simulated intestinal fluid (SIF) was prepared as follows: 218.7 g/L sodium chloride, 3.4 g/L sodium bicarbonate, 54 g/L bile salt, 36.7 g/L calcium chloride, 1 g/L bovine serum albumin, 24 g/L lipase, 24 g/L trypsin, adjusted to pH 7.0.

The experiments were performed with reference to Ma et al. [24]. Briefly, 15 g of the sample was placed in 15 mL of simulated saliva with 1 mol/L NaOH at a pH of 6.8, digested at a rate of 100 RPM /min for 5 min in a constant temperature shaker at 37 °C, and then 17.8 mL of simulated gastric juice was added and the pH was adjusted to 2.0 with 1 mol/L HCl. Digestion was performed at 100 RPM/min for 60 min on a constant temperature shaker at 37 °C. Finally, 23.4 mL of simulated intestinal fluid was added, and the pH was adjusted to 7.0 using 1 mol/L NaOH. Digestion was performed at 100 RPM/min on a constant-temperature shaker at 37 °C for 120 min. Then, the samples were transferred to a refrigerator at a temperature of −80 °C. In addition, artificial saliva, gastric juice, and intestinal juice were replaced with an equal volume of deionized water in the control sample (undigested SOB diluted sample).

### 2.13. Determination of Free Fatty Acids

With reference to Ding et al. [25], the pH-Stat method was used to quantify the free fatty acids (FFA) released during intestinal digestion (20, 40, 60, 80 and 120 min). Since the change in pH value during digestion was caused by the release of FFA, the quantification of FFA was performed by calculating the pH value of the NaOH neutralizing digestive fluid. The formula for the release percentage of FFA was as follows: where the pH value of yogurt was 7.0, V_1_ denotes the total volume of NaOH (μL), M_1_ denotes the molar concentration of NaOH (mol/L), M_L_ denotes the average molecular weight of oil (g/mol), and W_L_ denotes the total mass of oil (g).

$$\mathrm{FFA}\left(\%\right)=\left(\frac{V_{1}\times M_{1}\times M_{L}}{W_{L}}\right)\times 100$$

### 2.14. DPPH and ABTS Radical Scavenging Rate

These parameters were determined according to the method of Islam et al. [26] with appropriate modifications. First, 0.5 mL methanol extract was added to the DPPH solution (1.5 mL 0.2 mmol/L), and then reacted in darkness for 30 min after mixing. The absorbance value at 517 nm was measured using a spectrophotometer. Then 7.4 mmol/L ABTS solution (dissolved in methanol) was mixed with an equal volume of 2.6 mmol/L K2S2O8 solution (dissolved in methanol) and left to stand at room temperature in darkness for 12 h. After the reaction, the mixture was diluted to an appropriate percentage with methanol. The mixture was the ABTS working solution. Then, 0.2 mL methanol extract was added to 1.8 mL ABTS working solution, mixed well for 15 s and left to stand for 6 min. The absorbance value at 734 nm was determined using a spectrophotometer. The DPPH and ABTS radical scavenging rates of the sample were calculated according to the following formula: where A_i_ denotes the absorbance value of the sample reacting with ABTS and DPPH solution, A_j_ denotes the absorbance value of the sample reacting with methanol, and A_0_ denotes the absorbance value of the sample reacting with ABTS and DPPH solution.

$$\mathrm{Free}\;\mathrm{radical}\;\mathrm{clearance}\left(\%\right)=\left(1-\frac{A_{i}-A_{j}}{A_{0}}\right)\times 100$$

### 2.15. Determination of Protein Digestibility

Protein digestibility was determined according to the method of Li et al. [27]. First, 1 mL of gastric digestive juice, intestinal digestive juice, and undigested SOB yogurt samples were taken, and 1 mL of trichloroacetic acid (10%) was added and mixed, before being centrifuged (8000× *g*, 20 °C, 15 min) to obtain the supernatant, and the protein content in the samples was determined using the BCA method. The calculation formula is as follows: C_1_ represents the residual protein content in the supernatant, C_2_ represents the protein content of the control sample (undigested SOB yogurt).

$$\mathrm{Digestibility}\left(\%\right)=\frac{C_{1}}{C_{2}}\times 100$$

### 2.16. Determination of Sodium Dodecyl Sulphate-Polyacrylamide Gel Electrophoresis (SDS-PAGE)

The method of Nikiforidis et al. was employed, with some improvements [28]. After digestion, the supernatant sample was mixed with SDS-PAGE buffer (40% glycerol, 0.02% bromophenol blue, 0.25 mol/L Tris-HCl (pH 6.8 and 2% SDS)), boiled for 5 min, and separated proteins with 5% gel concentrate and 12% gel separation. Electrophoresis was performed in stacked gels at 80 V for 30 min and in separated gels at 120 V for 70 min. After electrophoresis, Coomassie bright blue R-250 was used for staining, and glacial acetic acid was used for decolorization.

### 2.17. Statistical Analysis

Five kinds of SOB yogurt with different contents were measured in this experiment. All results are presented as the averages of 3 random repeated measurements. The experimental data are expressed as mean ± standard deviation. SPSS 23.0 software was used to analyze the results of the data (ANOVA), and LSD was used to perform significance analysis; *p* < 0.05 represents statistically significant difference, *p* > 0.05 represents statistically insignificant difference, and Origin 2018 software was used for principal component analysis and mapping. Color difference linear expression: ΔE = [(ΔL*)^2^ + (Δa*)^2^ + (Δb*)^2^]^1/2^.

## 3. Results and Analysis

### 3.1. Physicochemical Properties of SOB Yogurt

Table 1 presents the physicochemical properties of yogurt with different substitutions of SOBs. The pH values of five kinds of yogurt showed a decreasing trend following 21 days of storage (*p* < 0.05), while the acidity of five kinds of yogurt increased and reached their maximum at 21 days. This could be related to the decomposition of lactose by bacterial metabolic activities during yogurt fermentation and storage, resulting in the production of lactic acid and other organic acids [29]. However, there was no significant difference in the pH and acidity of five kinds of yogurt during the same storage time (*p* > 0.05). This may be because the addition of SOBs has little effect on the pH and acidity of yogurt. 

In the same storage period, the water holding capacity of five kinds of yogurt was significantly different (*p* < 0.05). The yogurt with 25% substitution had the highest water holding capacity, while the yogurt with 100% substitution had the lowest water holding capacity. With the extension of storage time, the water holding capacity of the five yogurts decreased (*p* > 0.05). This may be because, with increasing SOB content, the surface charge and electrostatic repulsion increased, leading to whey separation. This is similar to the research results of Mantzouridou et al. [14]. 

There were significant differences in the protein content and solid content of the five kinds of yogurt (*p* < 0.05); the content of protein and solids in the yogurt with a substitution amount of 100% was the highest. There was no significant difference in fat content among the five kinds of yogurt (*p* > 0.05). This may be due to the fact that the SOBs contain 87–91.89% neutral fat droplets and 5.42–13% basic protein, and their protein content is higher than that of milk [11], thus increasing the content of protein and solids in the yogurt. 

Compared with the control group, a* and b * values increased and L* values decreased with increasing SOB substitution (*p* < 0.05); this is because the color of SOB itself makes the color of the yogurt dark. However, studies have shown that when the ΔE value reaches 3.7, the naked eye can perceive the difference in color [30]. Therefore, yogurt with SOBs instead of milk fat will not present unpleasant color characteristics to the naked eye.

### 3.2. Contents of Fatty Acids and Tocopherol in SOB Yogurt

As can be seen from Table 1, total fat content remained unchanged compared with the control group. The relative contents of saturated fatty acids (cardamic acid, palmitic acid, and stearic acid) in SOB yogurt decreased significantly with increasing milk fat substitution by SOBs (*p* < 0.05), and the relative contents of unsaturated fatty acids (oleic acid, linoleic acid, linolenic acid) were significantly increased (*p* < 0.05). This is mainly because the unsaturated fatty acids are mainly contained in the SOBs [31]. Studies have shown that large intake of cardamom acid can lead to plasma cholesterol and cardiovascular disease, while consumption of unsaturated fatty acids can improve high-density lipoprotein function and alleviate cardiovascular disease [32,33]. 

As shown in Table 1, no tocopherols were detected in the control group. The contents of δ-tocopherol, γ-tocopherol and α-tocopherol in SOB yogurt increased significantly with increasing milk fat substitution by SOBs (*p* < 0.05). This may be due to the large amount of tocopherol in SOBs [15]. The content of α-tocopherol was the highest, followed by δ-tocopherol and γ-tocopherol. Studies have shown that tocopherol can provide stable lipid products combining with lipid free radicals for hydrogen atoms and inhibit lipid chain oxidation [34,35]. In conclusion, increasing the substitution amount of SOBs replacing milk fat is conducive to increasing the tocopherol and fatty acid content in yogurt, thus increasing the antioxidant capacity and nutritional value of yogurt.

### 3.3. Texture Characteristics of SOB Yogurt

Table 2 shows the effect of replacing milk fat with SOBs on the texture characteristics of yogurt. The texture properties of yogurt in which 25% of the total fat was replaced were similar to those of control group. The hardness, consistency, cohesion, and viscosity index of the yogurt decreased with increasing SOB substitution (*p* < 0.05). This may be due to the decrease in casein content with increasing percentage of SOB replacement of milk fat, such that the protein gel network in yogurt was not able to wrap more fat globules [14].

### 3.4. Rheological Properties of SOB Yogurt

**Figure 1:**
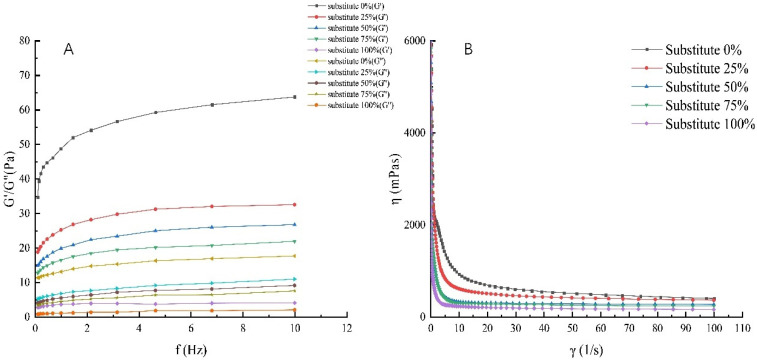
Effect of replacing milk fat by SOB on rheological properties of yogurt: (**A**) Curves of elastic modulus and viscous modulus of yogurt with different substitution amount; (**B**) The relationship between apparent viscosity and shear rate of yogurt with different substitution amount.

The stress generated by deformation is actually a kind of elastic energy stored in the sample, which is a measure of the elasticity of the sample, expressed by the elastic modulus G’. The amount of energy lost in the sample can be used to measure the viscosity of the sample, represented by the viscosity modulus G”. As shown in Figure 1A,B, when the total fat content remained unchanged, G’ and G” values decreased significantly with increasing milk fat substitution by SOBs (*p* < 0.05). At the same time, the apparent viscosity of the five yogurts decreased significantly with increasing shear rate, and all of them presented pseudoplastic fluid. The apparent viscosity of SOB yogurt was significantly lower than that of the control group (*p* < 0.05). This may be because of the decrease in casein quantity due to the increase in the amount of SOBs, the decrease in protein crosslinking degree, and the destruction of the protein gel network structure, resulting in decreased viscosity and elasticity [36]. As shown in Figure 1A, within the frequency range of 0.1–10 Hz, with increasing scanning frequency, the G’ and G” values of the five yogurts showed a rapidly increasing trend at first, followed by a slow increase, and the G’ values were all greater than the G” value, which may be due to the increase in the elastic percentage of the system by adding the SOB, so the samples showed solid-like characteristics [37].

### 3.5. PV and TBARS Values of SOB Yogurt

**Figure 2:**
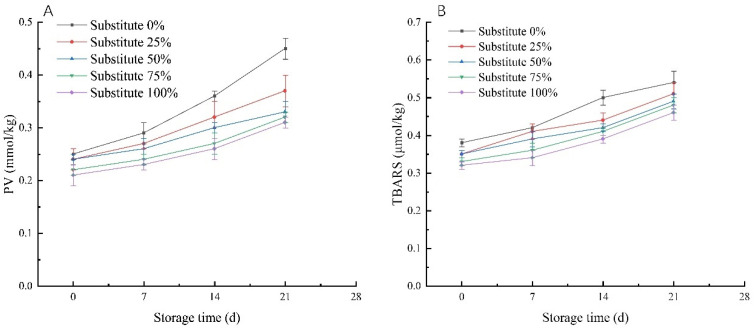
Effects of replacement of milk fat with SOBs on the PV value and TBARS value of yogurt: (**A**) PV value curves of yogurt with different substitution amounts; (**B**) TBARS value curves of yogurt with different substitution amounts.

As shown in Figure 2A,B, when the total fat content is kept unchanged, the PV and TBARS values of SOB yogurt show a significant rising trend with increasing storage time, and are significantly lower than those of the control group (*p* < 0.05). However, during the same storage time, with increasing milk fat substitution by SOBs, the PV and TBARS values of the SOB yogurt decreased significantly (*p* < 0.05). This may be due to the increased tocopherol content in the yogurt, which prevents the oxidation of polyunsaturated fatty acids in biofilms and cells and prevents the production of peroxide [38].

### 3.6. Microstructure of SOB Yogurt

**Figure 3:**
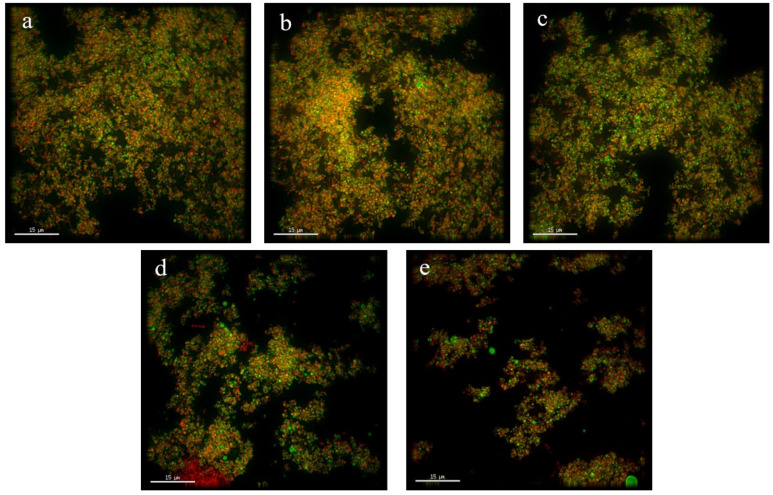
Microstructure of yogurt with different substitution amounts. Note: (**a**–**e**) are laser confocal micrographs of yogurt with 0% substitution, yogurt with 25% substitution, yogurt with 50% substitution, yogurt with 75% substitution and yogurt with 100% substitution, respectively.

Figure 3 shows the microstructure of the five kinds of yogurt prepared by substituting milk fat with SOBs at different substitution amounts. When the total fat content was kept unchanged, all the yogurts displayed a certain network structure. The yogurts in the control group and the yogurts with 25% and 50% substitution showed a highly crosslinked protein gel network, while the yogurts with 75% and 100% substitution showed a weakly crosslinked protein gel network. It can be seen that with increasing substitution of SOBs for milk fat, the degree of crosslinking of the protein gel network in the yogurt decreased significantly (*p* < 0.05). This may be because the spatial repulsion of κ-casein layer outside casein micelles gradually decreased during yogurt fermentation. When casein reaches the isoelectric point, casein micelles begin to aggregate with each other, forming a three-dimensional milk protein gel network through hydrophobic and electrostatic interactions that could not wrap more fat globules [39]. The numbers of fat globules visible in Figure 3a,b,c are significantly higher than those in Figure 3d,e, which may be due to the fact that more fat and protein are linked together and fused into the highly crosslinked protein gel structure, becoming an integral part of it.

### 3.7. Flavor of SOB Yogurt

**Figure 4:**
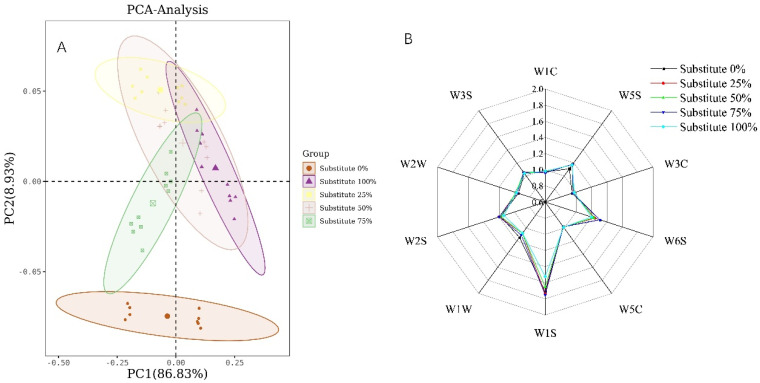
Effect of replacing milk fat with SOBs on the flavor of the yogurt: (**A**) PCA analysis of yogurt with different substitution amounts; (**B**) electronic nose radar diagram analysis of yogurt with different substitution amount.

**Figure 5:**
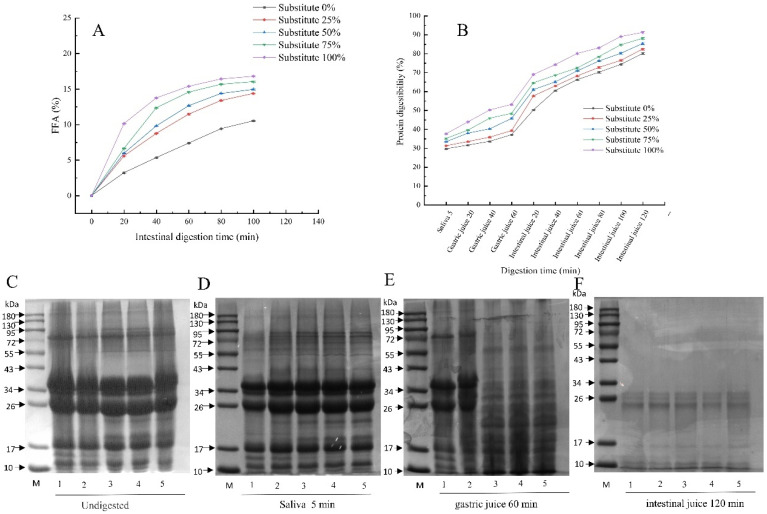
Effects of replacing milk fat with SOBs on the digestive characteristics of yogurt. Note: (**A**) Free fatty acids, (**B**) protein digestibility, (**C**) undigested, (**D**) saliva for 5 min, (**E**) artificial gastric juice digestion for 60 min, (**F**) artificial intestinal juice digestion for 120 min. M: Marker; Lanes 1–5 respectively represent substitution amount 0%, substitution amount 25%, substitution amount 50%, substitution amount 75%, substitution amount 100%.

When the total fat content is kept unchanged, the PCA analysis results of the five yogurts are shown in Figure 4A, in correlation matrix mode: The contribution rate of the first principal component is 86.853%, the contribution rate of the second principal component is 9.5454%, and the contribution rate of the two principal components is 96.38%, which is greater than 85%. Therefore, a two-dimensional diagram composed of these two principal components is able to represent the main information characteristics of the sample. Some regions of yogurt with 25%, 50%, 75% and 100% substitutions overlap, indicating that the overall flavor of the four yogurts is relatively close, but there are some differences. As shown in Figure 5B, there was significant difference between the control group and the SOB yogurt group (*p* < 0.05). The SOB yogurt showed the highest response in W1S, W6S, W5S, W2S and W3S, which may be due to the high content of short paraffins, such as methane, hydrogen, nitrogen oxides, and ethanol, and long paraffins.

### 3.8. Sensory Evaluation of SOB Yogurt

Each sensory evaluator scored the yogurt in terms of appearance, taste, bean smell, acidity, and overall evaluation, as shown in Table 3. When the total fat content remained unchanged, the apparent color of the yogurt in the control group was bright white. With increasing SOB substitution amount, the apparent color of the yogurt with a substitution amount of 25% was not significantly different from that in the control group (*p* > 0.05), which was significantly different from the other three kinds of yogurt (*p* < 0.05). Compared to the control group, the yogurt with 25% substitution had a thick, smooth taste and decreased sandy texture, while the gel strength of the other three yogurts was weak, with thin taste, lack of thickness, and bitter taste. Compared with the control group, the yogurts supplemented with SOBs had a bean smell, and the bean smell increased significantly with increasing SOB substitution amount (*p* < 0.05), but all within the acceptable range. There was no significant difference in the acidity of the five kinds of yogurt (*p* > 0.05). The yogurt with 25% substitution was white in color, thick and smooth in taste, with little bean smell and pleasant acidity, receiving the best overall evaluation.

### 3.9. Release Amount of Free Fatty Acids in SOB Yogurt

As shown in Figure 5A, when the total fat content was kept unchanged, The free fatty acid release curves of five kinds of yoghurt increased rapidly first and then slowly with the extension of digestion time. This may be due to the fact that trypsin and bile salts do not convert triacylglycerol to free fatty acids after 20 min digestion in simulated intestinal fluid. In the same digestion time, the release of free fatty acids in yogurt chyme increased with increasing SOB replacement of milk fat. This may be due to the small particle size of SOB yogurt, meaning that the same volume of fat will have a larger surface area, thus promoting lipase entering the oil-water interface and converting triglycerides into free fatty acids and glycerol, resulting in increased release of free fatty acids in digestive juices [40,41]. The free fatty acid release of yogurts with 25%, 50%, 75% and 100% substitution was significantly higher than that of the control group (*p* < 0.05). The results showed that with increasing replacement of milk fat by SOBs, the release of free fatty acids in the digestive process of yogurt increased, which is more conducive to the absorption of nutrients by the human body.

### 3.10. Protein Digestibility of SOB Yogurt

**Figure 6:**
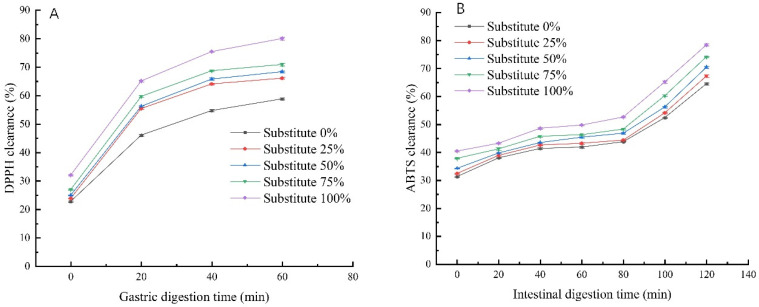
Effect of replacing milk fat with SOBs on antioxidant activity of yogurt: (**A**) DPPH clearance rate (**B**) ABTS clearance rate.

As shown in Figure 5B, the protein digestibility of SOB yogurt increased significantly with the extension of digestion time, and was higher than that of the control group (*p* < 0.05). The protein digestibility of yogurt in intestinal fluid is significantly higher than that in gastric fluid, which may be because trypsin in intestinal fluid is more likely to break peptide bonds than pepsin in gastric fluid [42]. This result is consistent with the results of protein molecular weight reduction after simulated gastric and intestinal digestion shown in Figure 6 After digestion, the protein digestibility of SOB yogurt was significantly higher than that of the control group (*p* < 0.05). This may be due to the small particle size of SOB yogurt, meaning that the contact surface area of the protein and protease on the droplet surface is larger, thus improving the digestibility of protein [40]. These results show that the protein digestibility of yogurt during digestion increased with increasing substitution amount of SOBs.

### 3.11. DPPH and ABTS Free Radical Scavenging Rates of SOB Yogurt

Figure 6 shows the influence of using different substitution amounts of SOBs to replace milk fat on the antioxidant capacity of the yogurt. As shown in Figure 6A,B, the DPPH and ABTS free radical scavenging rates of the SOB yogurt increased significantly with the extension of digestion time when the total fat content remained unchanged, and both were higher than those of the control group (*p* < 0.05). This may be due to the decrease in the average particle size of the SOB yogurt, resulting in decreased surface area and slowing down the oxidation rate [43]. In the same digestion time, the DPPH and ABTS free radical scavenging rates of SOB yogurt were significantly higher than those in the control group with increasing SOB replacement of milk fat. (*p* < 0.05). This may be because the content of tocopherol increases with increasing SOB content during digestion, which enhances antioxidant capacity. The DPPH free radical scavenging rate of the SOB yogurt increased significantly and then leveled off after 20 min of gastric juice digestion, which may be due to the further decrease in yogurt particle size after 5 min of oral digestion, and the interaction between SOBs and the yogurt protein network to form improved gel strength [14]. The ABTS free radical scavenging rate of SOB yogurt increased significantly during intestinal fluid digestion, which may be due to the fact that SOB yogurt is easy to digest and decompose with trypsin in intestinal fluid, which further reduces particle size and enhances antioxidant capacity.

## 4. Conclusions

When the total fat content of yogurt remains unchanged, the substitution of milk fat with different amounts of SOBs has a great influence on the functional characteristics, physicochemical properties, microstructure and other properties of the yogurt. With the increasing substitution of milk fat with SOBs, the pH and acidity of the yogurt changed little, but the physicochemical properties, protein gel network crosslinking degree, saturated fatty acid content, PV value and TBARS value of yogurt decreased significantly (*p* < 0.05). However, the protein content, solid content, unsaturated fatty acid content, tocopherol content and water holding capacity of yogurt increased significantly (*p* < 0.05). Compared with the control yogurt, the SOB yogurt had a significant difference in flavor (*p* < 0.05); however, the yogurt with 25% substitution had the highest sensory score. After digestion in vitro, the free fatty acid release, antioxidant capacity and protein digestibility of SOB yogurt were significantly increased (*p* < 0.05). The results of SDS-PAGE showed that the protein hydrolysis rate of yogurt with SOB was faster. In conclusion, yogurt with 25% substitution had the best quality and functional characteristics, and the results of this experiment can lay the foundation for further development and utilization of SOBs.

## Figures and Tables

**Table 1:** Physicochemical properties of yogurt with different substitution amounts of SOBs replacing milk fat.

Parameter	Days	Substitution Amount				
		0%	25%	50%	75%	100%
pH value	0	4.50 ± 0.02 ^a^	4.49 ± 0.05 ^a^	4.48 ± 0.04 ^a^	4.49 ± 0.03 ^a^	4.48 ± 0.01 ^a^
	7	4.43 ± 0.04 ^a^	4.42 ± 0.03 ^a^	4.41 ± 0.06 ^a^	4.42 ± 0.03 ^a^	4.44 ± 0.02 ^a^
	14	4.32 ± 0.04 ^a^	4.31 ± 0.02 ^a^	4.34 ± 0.04 ^a^	4.32 ± 0.02 ^a^	4.31 ± 0.02 ^a^
	21	4.08 ± 0.03 ^a^	4.06 ± 0.02 ^a^	4.09 ± 0.04 ^a^	4.07 ± 0.04 ^a^	4.05 ± 0.01 ^a^
Titratable acidity (°T)	0	65.62 ± 0.16 ^a^	67.34 ± 0.13 ^a^	67.14 ± 0.08 ^a^	69.16 ± 0.17 ^a^	65.98 ± 0.13 ^a^
	7	73.66 ± 0.09 ^a^	72.83 ± 0.15 ^a^	74.71 ± 0.11 ^a^	74.35 ± 0.12 ^a^	73.63 ± 0.10 ^a^
	14	80.15 ± 0.18 ^a^	81.03 ± 0.14 ^a^	79.13 ± 0.22 ^a^	79.75 ± 0.12 ^a^	80.15 ± 0.21 ^a^
	21	90.34 ± 0.16 ^a^	89.76 ± 0.20 ^a^	90.98 ± 0.18 ^a^	90.19 ± 0.14 ^a^	89.83 ± 0.17 ^a^
water-holding power (%)	0	65.57 ± 0.06 ^d^	88.57 ± 0.27 ^a^	76.18 ± 0.17 ^b^	72.77 ± 0.10 ^c^	59.41 ± 0.12 ^e^
	7	56.16 ± 0.11 ^c^	86.42 ± 0.15 ^a^	68.11 ± 0.05 ^b^	66.92 ± 0.03 ^b^	47.46 ± 0.08 ^d^
	14	53.46 ± 0.10 ^c^	82.60 ± 0.08 ^a^	63.53 ± 0.14 ^b^	64.35 ± 0.12 ^b^	36.67 ± 0.15 ^d^
	21	47.67 ± 0.13 ^c^	78.43 ± 0.15 ^a^	59.32 ± 0.12 ^b^	58.76 ± 0.04 ^b^	32.45 ± 0.08 ^d^
Protein content (%)		2.97 ± 0.03 ^e^	3.17 ± 0.03 ^d^	3.34 ± 0.03 ^c^	3.58 ± 0.04 ^b^	3.76 ± 0.04 ^a^
Fat content (%)		3.54 ± 0.02 ^a^	3.54 ± 0.03 ^a^	3.55 ± 0.03 ^a^	3.56 ± 0.04 ^a^	3.55 ± 0.04 ^a^
Solid content (%)		15.47 ± 0.04 ^e^	15.68 ± 0.03 ^d^	15.83 ± 0.07 ^c^	16.10 ± 0.03 ^b^	16.26 ± 0.05 ^a^
Color						
L*		102.55 ± 0.04 ^a^	98.28 ± 0.11 ^b^	94.46 ± 0.06 ^c^	91.91 ± 0.13 ^d^	90.73 ± 0.18 ^d^
a*		−1.31 ± 0.06 ^b^	−1.20 ± 0.04 ^b^	−0.82 ± 0.08 ^a^	−0.52 ± 0.08 ^a^	−0.47 ± 0.04 ^a^
b*		7.62 ± 0.08 ^e^	9.36 ± 0.01 ^d^	10.49 ± 0.08 ^c^	11.41 ± 0.08 ^b^	12.35 ± 0.05 ^a^
ΔE			0.11	0.76	1.52	2.33
Fatty acids						
Cinnamic acid		12.74 ± 0.05 ^a^	7.96 ± 0.01 ^b^	4.22 ± 0.02 ^c^	2.22 ± 0.00 ^d^	0.93 ± 0.01 ^e^
Palmitic acid		43.67 ± 0.14 ^a^	31.78 ± 0.08 ^b^	22.33 ± 0.07 ^c^	17.29 ± 0.04 ^d^	14.02 ± 0.03 ^e^
Stearic acid		13.76 ± 0.08 ^a^	10.31 ± 0.02 ^b^	7.44 ± 0.02 ^c^	5.95 ± 0.03 ^d^	5.06 ± 0.01 ^e^
Oleic acid		25.36 ± 0.13 ^e^	26.32 ± 0.12 ^d^	26.95 ± 0.08 ^c^	27.60 ± 0.04 ^b^	28.01 ± 0.11 ^a^
Linoleic acid		4.08 ± 0.03 ^e^	20.68 ± 0.07 ^d^	34.30 ± 0.10 ^c^	41.30 ± 0.12 ^b^	45.78 ± 0.08 ^a^
Linolenic acid		0.39 ± 0.01 ^e^	2.95 ± 0.01 ^d^	4.76 ± 0.03 ^c^	5.64 ± 0.02 ^b^	6.20 ± 0.02 ^a^
Saturated fatty acid		70.17 ± 0.06 ^a^	50.05 ± 0.07 ^b^	33.99 ± 0.03 ^c^	25.46 ± 0.08 ^d^	20.01 ± 0.05 ^e^
Unsaturated fatty acid		29.83 ± 0.02 ^e^	49.95 ± 0.12 ^d^	66.01 ± 0.11 ^c^	74.54 ± 0.12 ^b^	79.99 ± 0.17 ^a^
Tocopherol (µg/kg)						
δ-tocopherol		-	517.11 ± 1.47 ^d^	1203.32 ± 2.41 ^c^	1558.99 ± 7.16 ^b^	2223.31 ± 10.61 ^a^
γ-tocopherol		-	201.68 ± 1.29 ^d^	275.64 ± 1.68 ^c^	326.42 ± 1.57 ^b^	450.66 ± 1.72 ^a^
α-tocopherol		-	862.16 ± 1.45 ^d^	1415.29 ± 4.13 ^c^	1986.62 ± 7.89 ^b^	2829.54 ± 9.71 ^a^

Note: Results are mean ± SD of three determinations. Different letters in the upper right corner of peer data (a, b, c, d, e) indicate significant differences (*p* < 0.05); The same letters indicate no significant difference (*p* > 0.05), the same as in the following table.

**Table 2:** Texture of yogurt with different substitution amounts of SOB replacing milk fat.

Indicators	0%	25%	50%	75%	100%
Hardness (g)	119.20 ± 0.83 ^a^	113.56 ± 0.49 ^b^	87.24 ± 0.39 ^c^	63.02 ± 0.34 ^d^	48.37 ± 0.96 ^e^
Consistency (g·s)	1060.04 ± 1.54 ^a^	1027.31 ± 2.26 ^a^	733.43 ± 1.68 ^b^	543.60 ± 1.33 ^c^	413.94 ± 0.69 ^d^
Cohesion (g)	50.11 ± 0.98 ^a^	48.65 ± 0.98 ^a^	38.41 ± 0.71 ^b^	30.17 ± 0.75 ^c^	22.23 ± 0.70 ^d^
Viscosity index (g·s)	500.41 ± 0.83 ^a^	472.76 ± 0.20 ^a^	359.46 ± 0.29 ^b^	250.98 ± 0.14 ^c^	143.97 ± 0.36 ^d^

different letters (a, b, c, d, e) in the upper right corner of peer data indicate significant differences (*p* < 0.05); The same letter means no significant difference (*p* > 0.05).

**Table 3:** Sensory evaluation of yogurt with different substitution amount.

Indicators	0%	25%	50%	75%	100%
apparent	8.42 ± 0.23 ^a^	8.33 ± 0.20 ^a^	7.79 ± 0.18 ^b^	7.40 ± 0.22 ^c^	7.23 ± 0.23 ^d^
taste	8.17 ± 0.16 ^b^	8.41 ± 0.15 ^a^	7.60 ± 0.23 ^c^	6.77 ± 0.14 ^d^	6.37 ± 0.21 ^e^
bean smell	0.00 ± 0.00 ^e^	0.31 ± 0.02 ^d^	0.45 ± 0.03 ^c^	0.65 ± 0.04 ^b^	1.01 ± 0.04 ^a^
acidity	8.36 ± 0.23 ^a^	8.38 ± 0.16 ^a^	8.31 ± 0.21 ^a^	8.37 ± 0.22 ^a^	8.35 ± 0.26 ^a^
overall evaluation	8.27 ± 0.14 ^b^	8.54 ± 0.29 ^a^	7.77 ± 0.31 ^c^	7.23 ± 0.27 ^d^	6.78 ± 0.16 ^e^

different letters (a, b, c, d, e) in the upper right corner of peer data indicate significant differences (*p* < 0.05); The same letter means no significant difference (*p* > 0.05).

## Data Availability

Data is contained within the article.
